# Combined Arterial Reconstruction and Surgical Distal Venous Arterialization for Limb Salvage in Thromboangiitis Obliterans: A Case Report

**DOI:** 10.70352/scrj.cr.25-0342

**Published:** 2025-07-29

**Authors:** Yuri Yoshida, Shinsuke Kikuchi, Daichi Mizushima, Hirofumi Jinno, Hiroya Moriyama, Takayuki Uramoto, Kazuki Takahashi, Tsutomu Doita, Keisuke Kamada, Seima Ohira, Daiki Uchida, Naoya Kuriyama, Nobuyoshi Azuma

**Affiliations:** 1 Department of Vascular Surgery, Asahikawa Medical University, Asahikawa, Hokkaido, Japan; 2 Department of Cardiovascular Surgery, Sapporo Kosei General Hospital, Sapporo, Hokkaido, Japan; 3 Department of Vascular Surgery, Asahikawa City Hospital, Asahikawa, Hokkaido, Japan; 4 Department of Cardiovascular Surgery, Osaka University Graduate School of Medicine, Suita, Osaka, Japan

**Keywords:** thromboangiitis obliterans, surgical distal venous arterialization, skin perfusion pressure

## Abstract

**INTRODUCTION:**

Thromboangiitis obliterans (TAO) has become increasingly uncommon in Japan due to declining smoking prevalence. However, in advanced cases with severely compromised distal vasculature, achieving durable limb salvage remains a formidable surgical challenge.

**CASE PRESENTATION:**

A 51-year-old man with a 12-year history of TAO presented with rest pain and a necrotic ulcer on the 2nd toe. He had recently ceased smoking after a 31-year history. Imaging demonstrated complete occlusion of the popliteal and tibial arteries, with foot perfusion reliant on corkscrew collaterals. The ankle-brachial index was 0.43, and skin perfusion pressure (SPP) was critically low. A severely diseased plantar artery was identified as a potential distal target. Given the high risk of graft failure, a hybrid strategy combining *in situ* bypass and surgical distal venous arterialization (DVA) was preoperatively planned. To mitigate perioperative vasospasm, a lumbar sympathetic block was administered 1 week prior to surgery. An *in situ* bypass using the ipsilateral great saphenous vein was constructed from the superficial femoral artery to the plantar artery. DVA was established via retrograde puncture of the plantar vein, balloon angioplasty for valve sites, and end-to-side anastomosis to the bypass graft. Early duplex ultrasonography revealed anastomotic stenosis at the DVA site as well as stenosis at valve sites, both of which were successfully managed with a single endovascular procedure. The toe stump healed completely within 3 months. The graft remained patent for 2 years, and SPP was preserved even after graft occlusion. Notably, graft failure coincided with DVA occlusion, suggesting its critical role in maintaining flow. At 42 months postoperatively, the patient remained ulcer-free with favorable perfusion, pain-free ambulation, and full return to work.

**CONCLUSIONS:**

Preoperatively planned surgical DVA, in conjunction with sympathetic modulation and timely postoperative intervention, may offer a durable limb salvage strategy in advanced TAO with limited distal targets.

## Abbreviations

DVA
: distal venous arterialization
GSV
: great saphenous vein
SPP
: skin perfusion pressure
TAO
: thromboangiitis obliterans

## INTRODUCTION

The incidence of TAO, also known as Buerger’s disease, has significantly declined in Japan due to widespread smoking cessation.^1)^ Consequently, cases requiring surgical revascularization have become increasingly rare.^2,3)^ However, in patients with anatomically complex disease where conventional arterial reconstruction is not feasible, innovative surgical approaches remain essential for successful limb salvage in advanced cases of TAO.

We report a rare case of critical limb ischemia due to TAO, characterized by the absence of reconstructable distal arteries and accompanied by severe ischemic pain and digital ulceration. Limb salvage was successfully achieved through a combined approach of arterial reconstruction and DVA, a technique known as surgical DVA, in which arterial blood is rerouted into the venous system of the foot to perfuse ischemic tissue.^4)^ This case highlights the technical considerations and surgical ingenuity required in managing such challenging vascular anatomy, and underscores the potential of DVA as a viable limb-saving option in select patients.

## CASE PRESENTATION

**Fig. 1:**
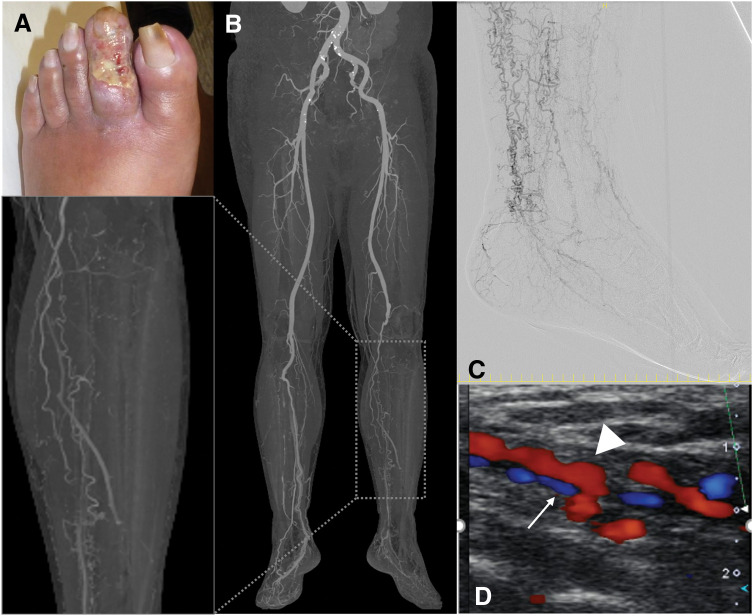
Clinical presentation and preoperative evaluation. Left 2nd toe ulcer observed at the time of admission (**A**). CTA demonstrated occlusion distal to the left popliteal artery, with foot perfusion maintained via collateral circulation (**B**). Digital subtraction angiography revealed the absence of normal arteries in the foot, with the presence of corkscrew-like collateral vessels (**C**). Preoperative ultrasonography confirmed the patency of the plantar arteries and veins. The arrow indicates the plantar artery, and the arrowhead indicates the plantar vein. Despite the small vessel diameters, a potential site for anastomosis was identified (**D**).

A 51-year-old man, previously diagnosed with TAO 12 years prior and managed conservatively with pharmacotherapy, presented to our department for limb salvage as a 2nd opinion after being advised to undergo major amputation. He had developed resting pain and a skin ulcer on the 2nd toe of his left foot. The patient had a 31-year history of smoking 15 cigarettes per day but successfully ceased smoking following cessation counseling. He had comorbid hypertension and dyslipidemia, but no other significant atherosclerotic risk factors. On presentation, the left 2nd toe exhibited a painful, infected ulcer (**Fig. 1A**). The ankle-brachial index was 0.43, and SPP was severely reduced at 12 mmHg on the dorsum and 20 mmHg on the plantar aspect of the foot. CTA revealed complete occlusion of the mid-portion of the left popliteal artery, with collateral perfusion of the lower leg via genicular arteries. All tibial arteries were occluded, and extensive corkscrew-shaped collateral vessels were observed (**Fig. 1B**). Digital subtraction angiography confirmed the absence of native arterial flow to the foot, with perfusion entirely dependent on tortuous collateral vessels, consistent with a so-called “desert foot” (**Fig. 1C**). Despite the poor vascular status, we initiated treatment with the goal of wound healing through revascularization, emphasizing strict smoking cessation. Endovascular therapy was deemed unsuitable due to the high risk of occluding the few patent collaterals and the absence of targetable native arteries. Ultrasonography identified a pinpoint anastomosis site on the plantar artery, with an external diameter of 1.1 mm and a luminal diameter of 0.8 mm, accompanied by significant wall thickening and poor visualization of the medial and lateral plantar branches (**Fig. 1D**). Given the high risk of early graft failure, we opted to perform surgical DVA to maintain graft flow.

Preoperative ultrasound vein mapping confirmed that the ipsilateral GSV, with a consistent diameter >3.0 mm along its course, was suitable for use. To mitigate the risk of arterial spasm associated with surgical manipulation, a lumbar sympathetic block was administered 1 week preoperatively by an anesthesiologist. Under general anesthesia and in the supine position, an *in situ* bypass was constructed using the GSV, with the proximal anastomosis to the superficial femoral artery and the distal anastomosis to the plantar artery. An 8-0 polypropylene suture was used for the distal anastomosis, employing a continuous suture technique initiated from the heel side, with three-point fixation at the toe. Although the plantar artery was significantly thickened, successful anastomosis was achieved. A 2 mm arteriotomy cannula (Medtronic, Minneapolis, MN, USA) was inserted via a branch of the GSV, allowing sequential intra-graft infusion of a vasodilator cocktail—comprising 2.5 µg prostaglandin E_1_, 1.25 mg isosorbide dinitrate, and 2.5 mg milrinone. Graft flow, initially measured at 1 mL/min, improved to 6 mL/min following administration, as assessed using the VeriQ C system (Medistim, Oslo, Norway).

**Fig. 2:**
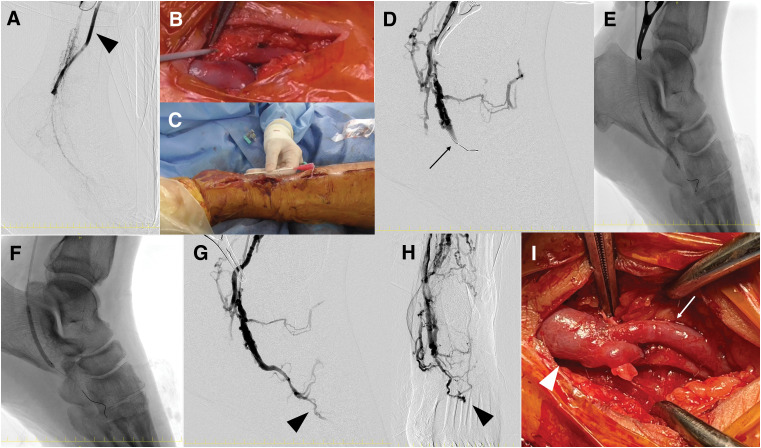
Intraoperative findings of plantar artery reconstruction and surgical DVA. Completion angiography following left superficial femoral artery to common plantar artery bypass. The arrowhead denotes the vein graft (**A**). To initiate surgical DVA, an 18-gauge cannula was inserted into one of the accompanying plantar veins (**B**). A 4 Fr sheath was then retrogradely advanced into the foot (**C**). Intraoperative venography revealed a competent venous valve (arrow), impeding retrograde contrast flow (**D**). The distal valve was successfully disrupted using a 3 mm balloon (**E**). The proximal valve was subsequently dilated with a 4 mm balloon (**F**). Post-dilation venography confirmed adequate retrograde opacification extending to the metatarsal vein (arrowhead), indicating successful valve disruption (**G** and **H**). The sheath was removed, and the treated plantar vein (arrow) was anastomosed end-to-side to the vein graft (arrowhead), thereby completing the surgical DVA (**I**). DVA, distal venous arterialization

Angiography demonstrated corkscrew-like arterial flow, particularly in the plantar artery, which served as the distal runoff vessel (**Fig. 2A**). A retrograde puncture of the plantar vein accompanying the anastomosed artery was performed, and a 0.035-inch guidewire was advanced through a 4 Fr introducer IIH sheath (10 cm in length; Terumo, Tokyo, Japan, **Fig. 2B**, **2C**). A Chevalier 14 Universal guidewire (Nipro, Osaka, Japan) was advanced to the medial plantar vein. After confirming the location of venous valves via contrast injection (**Fig. 2D**), balloon angioplasty was performed using a 3.0 × 40 mm Coyote balloon (Boston Scientific, Marlborough, MA, USA) at 14 atm for 60 s, repeated 3 times, to achieve retrograde perfusion to the distal medial plantar vein (**Fig. 2E**). Additional angioplasty was performed at the proximal medial and common plantar venous valves using a 4.0 × 40 mm Shiden balloon (Kaneka, Osaka, Japan) at 14 atm for 60 s, repeated three times (**Fig. 2F**). Visualization of the metatarsal veins confirmed successful venous arterialization (**Fig. 2G**, **2H**). The plantar vein was then anastomosed end-to-side to the graft using an 8-0 polypropylene suture, employing a continuous suturing technique initiated from the heel side (**Fig. 2I**). The final graft flow was measured at 190 mL/min using transit time flow measurement with the VeriQ C system (Medistim). The 2nd toe was amputated at the metatarsophalangeal joint. Total operative time was 5 hours and 27 minutes.

**Fig. 3:**
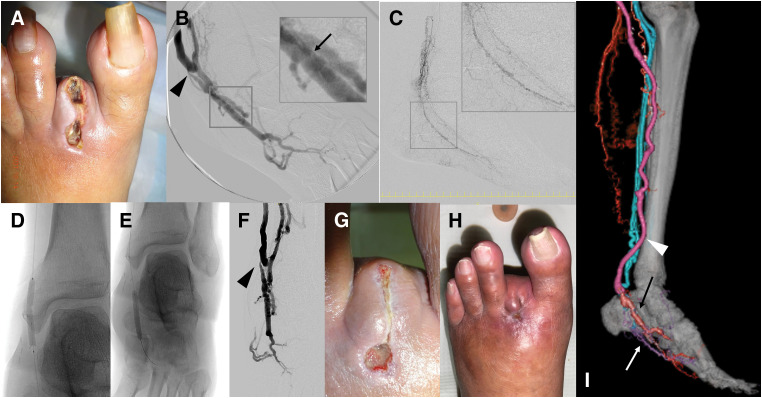
Postoperative course and intervention following surgical DVA. At 2 weeks postoperatively, the stump of the left 2nd toe exhibited no evidence of epithelialization; however, local infection remained well controlled (**A**). Angiographic evaluation revealed a focal stenosis at the DVA anastomotic site (arrowhead) and a suspected venous valve (arrow) impeding retrograde flow (**B**). At 1 month, endovascular reintervention was undertaken. A microcatheter was advanced to the plantar artery anastomosis, and contrast injection delineated the plantar artery via the diseased venous segment (**C**). Balloon angioplasty was performed using a 3 mm balloon to address both the anastomotic narrowing and the valve-related stenosis (**D, E**). Despite only modest angiographic improvement at the anastomotic site (arrowhead), venous opacification via the DVA was markedly augmented, indicating effective distal perfusion (**F**). At 2 months, the toe stump demonstrated progressive reduction in size (**G**). Complete wound healing was achieved by 3 months postoperatively (**H**). One-year follow-up CTA confirmed sustained patency of the vein graft (arrowhead), with clear delineation of the DVA (black arrow) and the plantar artery (white arrow) (**I**). DVA, distal venous arterialization

At 3 weeks postoperatively, duplex ultrasonography revealed stenosis with accelerated flow at the DVA anastomosis and additional valve-related stenosis distally (**Table 1**). There was still no sign of healing at the stump of the 2nd toe, but the skin redness has improved (**Fig. 3A**). Under left lower limb nerve block anesthesia, a 2 mm arteriotomy cannula was inserted via a branch of the vein graft to perform angiography during ligation of the arteriovenous fistula following *in situ* bypass. DVA flow was observed extending to the metatarsal region (**Fig. 3B**), and the venous valve site previously noted on duplex ultrasonography was successfully identified on angiography as well (**Fig. 3B**). One month postoperatively, endovascular intervention was performed via antegrade access through the left common femoral artery using a 3 Fr ParentPlus sheath (50 cm in length; Medikit, Tokyo, Japan). A Jupiter FC3 Peripheral Guidewire (Boston Scientific) was advanced to the plantar artery anastomosis, and a Prominent Advance Standard microcatheter (135 cm in length; Tokai Medical Products, Aichi, Japan) was positioned to evaluate perfusion of the foot artery (**Fig. 3C**). The guidewire was advanced to the DVA anastomosis using the microcatheter to facilitate crossing of the DVA. Balloon angioplasty was performed at the anastomotic and valve stenosis sites using a 3.0 × 40 mm Coyote balloon (Boston Scientific), with inflation at 6 atm for 120 s and at 14 atm for 60 s, each repeated twice, respectively (**Fig. 3D**–**3F**).

**Table 1:** Postoperative changes in skin perfusion pressure of the index limb and duplex ultrasonographic evaluation of vein graft, distal venous arterialization, and plantar artery

Measurement	2 W	3 W	4 W	6 W	2 M	4 M	9 M	12 M	18 M	24 M	36 M	42 M
SPP (mmHg)												
Dorsum	16	–	–	–	17	16	30	36	–	33	31	35
Plantar surface	30	–	–	–	38	31	38	46	–	58	56	16
DUS (Vein graft)												
PSV	40	40	69	58	66	52	68	43	40	49	–	–
Flow	282	305	580	550	538	770	758	641	432	359	–	–
Diameter	4.7	4.7	5	5.3	4.9	5.3	6	6.7	6.5	6.3	–	–
DUS (DVA anastomosis site)												
PSV	–	609	603	632	693	518	347	236	239	150	–	–
Diameter	–	1.0	1.1	1	0.9	2.2	2	2.1	1.9	1.8	–	–
DUS (Venous valve site)												
PSV	–	284	303	527	267	156	539	518	265	118	–	–
DUS (Plantar artery)												
Flow	–	–	–	–	–	–	48	53	57	–	–	–

Units are expressed as follows: PSV (cm/s), Diameter (mm), and Flow (mL/min).

DUS, duplex ultrasonography; M, month; PSV, peak systolic velocity; SPP, skin perfusion; pressure; W, week

At 2 months postoperatively, the plantar SPP had improved to 38 mmHg, and the 2nd toe stump showed signs of healing with a reduction in wound size (**Fig. 3G**). By 3 months, the stump had completely healed (**Fig. 3H**). Graft flow peaked at 1 year postoperatively and gradually declined thereafter (**Table 1**). Although the DVA anastomotic diameter had increased, the observed reduction in graft flow is most likely attributable to restenosis at the distal venous valve site. By 9 months after surgery, flow in the plantar artery had become detectable. One year after surgery, CTA shows that the vein graft remains well-patent, with the reconstructed plantar artery and the arterialized plantar vein clearly visualized (**Fig. 3I**). With sustained smoking cessation, the SPP rose above 50 mmHg. As the patient remained asymptomatic, no additional intervention was undertaken despite the observed reduction in graft flow. Graft patency was last confirmed via duplex ultrasonography at 24 months postoperatively, with occlusion noted thereafter. SPP was monitored longitudinally to evaluate postoperative limb perfusion. At 36 months, values remained favorable at 31 mmHg on the dorsal aspect and 56 mmHg on the plantar surface, and no ulcer recurrence was observed. At 42 months, however, SPP had declined to 35 mmHg dorsally and 15 mmHg on the plantar aspect. Despite this decrease, the patient continued to abstain from smoking and remained free of rest pain throughout the follow-up period (**Table 1**).

## DISCUSSION

This case highlights the successful limb salvage and social reintegration of a patient with advanced TAO through a preoperatively planned combination of surgical DVA and arterial bypass. In TAO, where distal arterial reconstruction is often anatomically unfeasible due to diffuse occlusions and poor vessel quality, conventional revascularization strategies are frequently limited.^4)^ In this patient, the plantar artery was severely diseased and barely reconstructable, prompting the decision to combine *in situ* bypass with surgical DVA to maintain graft patency. While an arteriovenous fistula might have sufficed for graft flow preservation, DVA was selected for two key reasons: (1) to enhance oxygen delivery to the ischemic foot under severely restricted arterial inflow, and (2) to mitigate excessive venous return and associated edema.^5)^ However, it is important to recognize the functional limitations of DVA. In this case, angiographic findings revealed that DVA perfusion did not reach the level of the ischemic toe (**Figs. 2H** and **3B**), suggesting that DVA alone would have been insufficient for wound healing. Therefore, arterial reconstruction, even with diseased vessels, remains fundamental when targeting digital ulcers.

A major technical challenge in surgical DVA is the effective destruction of venous valves to ensure adequate retrograde perfusion. Currently, no dedicated devices exist for retrograde valve disruption in the pedal veins, and tools such as balloon cutters, probes, surgical dilators, and Fogarty catheters are used instead.^4,6,7)^ In this case, restenosis at the valve sites was observed after 1 year, leading to decreased graft flow. This suggests that incomplete valve destruction was a critical limitation of the procedure (**Table 1**). Nevertheless, the relatively large diameter of the plantar venous system may have contributed to prolonged graft patency. Multiple studies have highlighted the limited durability of graft patency following surgical DVA, despite its potential as a limb-salvaging strategy for patients with no-option chronic limb-threatening ischemia. Lu et al. reported a 1-year limb salvage rate of 71%, yet the secondary patency rate was only 46%, indicating a high rate of graft failure or reintervention.^8)^ Similarly, Schreve et al. found 1-year patency rates ranging from 59% to 71%, with a further decline of approximately 10% by the 2nd year.^9)^ Ho et al. reported comparable outcomes and emphasized the considerable heterogeneity in surgical techniques, which complicates direct comparisons and may contribute to inconsistent patency outcomes.^10)^ Collectively, these findings underscore a critical limitation of surgical DVA: while it may offer a last-resort option for limb salvage, the consistently suboptimal graft patency rates raise significant concerns regarding its long-term efficacy and sustainability.

Regarding wound healing, Sasajima et al. reported a 67% healing rate with surgical DVA when combined with arterial bypass and free tissue transfer,^4)^ while Mutirangura et al. observed a 73% healing rate using DVA with bypass.^11)^ By contrast, outcomes with surgical DVA alone were notably inferior: Sasajima’s series reported a healing rate of only 50%, and in Mutirangura’s study, 23% of non-healed cases ultimately required major amputation. These findings underscore the critical importance of adjunctive procedures—such as bypass surgery and tissue reconstruction—in achieving successful wound healing, particularly in patients with extensive tissue loss. In our case, early graft stenosis was successfully managed with a single endovascular intervention, and graft patency was maintained for 2 years. Following DVA occlusion, the bypass graft also thrombosed, suggesting that the DVA played a pivotal role in sustaining graft flow. Although angiographic findings indicated that revascularization of the plantar artery contributed more directly to the healing of the 2nd toe, the patency of the bypass graft was dependent on the DVA circuit.

This case illustrates the complexity of managing TAO-related limb ischemia, where multiple therapeutic strategies may collectively influence clinical outcomes. In addition to the surgical approach combining plantar artery bypass and DVA, the patient’s cessation of smoking, preoperative sympathetic nerve block, intra-graft administration of vasodilators, and partial toe amputation likely contributed to tissue perfusion and ulcer healing. While it is not feasible to isolate the effects of each intervention within a single case, this multimodal approach reflects common clinical practice in the treatment of critical limb ischemia in TAO patients. Despite the observed decrease in SPP, the patient remained free of rest pain and continued smoking cessation, indicating clinical stability. The marked decline in plantar SPP following graft occlusion suggests that graft patency may have played a role in maintaining tissue perfusion in the reconstructed plantar arterial territory. This case underscores the potential value of combining bypass and DVA as part of a comprehensive revascularization strategy, while also highlighting the need for future observational studies to better define the relative contributions and synergies of adjunctive therapies in TAO.

## CONCLUSIONS

This case demonstrates that even in TAO patients with severely limited distal arterial targets, a planned combination of arterial bypass and surgical DVA can achieve durable limb salvage and functional recovery. Further refinement of valve disruption techniques and standardization of surgical DVA procedures are essential to improve long-term outcomes.

## ACKNOWLEDGMENTS

This study was supported by Asahikawa Medical University Surgical Educational Support Organization (AMUSE).

## DECLARATIONS

### Funding

None.

### Authors’ contributions

YY, SK, DM, HM, DU, TD, and NA managed the perioperative course.

YY, SK, HJ, TU, TD, KT, KK, SO, and NK followed the patient at the outpatient clinic.

YY, SK, and NA made the critical revision.

YY, SK, DM, and HM managed wound care.

YY and SK wrote the manuscript.

All the authors read and approved the final manuscript.

### Availability of data and materials

The data used for this case report are available from the corresponding author upon request.

### Ethics approval and consent to participate

This work does not require ethical considerations or approval. Informed consent to participate in this study was obtained from the patient.

### Consent for publication

Informed consent for publication of this case report was obtained from the patient.

### Competing interests

The authors declare that they have no competing interests.
